# Principles and Applications of Loop-Mediated Isothermal Amplification to Point-of-Care Tests

**DOI:** 10.3390/bios12100857

**Published:** 2022-10-10

**Authors:** Jee-Woong Park

**Affiliations:** Medical Device Development Center, Daegu-Gyeongbuk Medical Innovation Foundation (K-MEDI Hub), Daegu 41061, Korea; jp@kmedihub.re.kr

**Keywords:** LAMP, molecular diagnosis, isothermal amplification, point of care

## Abstract

For the identification of nucleic acids, which are important biomarkers of pathogen-mediated diseases and viruses, the gold standard for NA-based diagnostic applications is polymerase chain reaction (PCR). However, the requirements of PCR limit its application as a rapid point-of-care diagnostic technique. To address the challenges associated with regular PCR, many isothermal amplification methods have been developed to accurately detect NAs. Isothermal amplification methods enable NA amplification without changes in temperature with simple devices, as well as faster amplification times compared with regular PCR. Of the isothermal amplifications, loop-mediated isothermal amplification (LAMP) is the most studied because it amplifies NAs rapidly and specifically. This review describes the principles of LAMP, the methods used to monitor the process of LAMP, and examples of biosensors that detect the amplicons of LAMP. In addition, current trends in the application of LAMP to smartphones and self-diagnosis systems for point-of-care tests are also discussed.

## 1. Introduction

Nucleic acids (NAs) are important biomarkers of pathogen-mediated diseases and viruses. The identification of NAs using amplification strategies has been effectively utilized in diagnostic methods in the treatment of a variety of infectious diseases for the accurate detection and identification of the causative organism.

The gold standard for NA-based diagnostic applications is polymerase chain reaction (PCR), which specifically amplifies target-DNA sequences in an exponential manner.

As the gold standard for molecular diagnosis, PCR-based POC techniques are frequently developed and commercialized [1,2]. Cepheid Xpert Xpress SARS-CoV-2/Flu/RSV, which was approved by the FDA for emergency use, showed a limit of detection as low as 100 viral copies/mL [3]. The Visby Medical RT-PCR Portable Device, designed for portable and easy-to-use RT-PCR devices, showed that its clinical sensitivity is 95% and that its specificity is 100% for SARS-CoV-2 [4]. The VitaPCR^TM^ RT-PCR assay showed 99.3% sensitivity and 94.7% specificity for SARS-CoV-2 [5]. 

However, the requirement of an expensive and sophisticated thermocycler, trained technical personnel, and extended reaction times limits the application of PCR-based POC techniques in rapid point-of-care diagnosis.

The other drawback is the non-specific annealing of primers and false positives, which further delay the development of PCR-based point-of-care (POC) diagnostics [6,7].

To address the challenges associated with regular PCR, many isothermal amplification methods have been developed to accurately detect NAs [8,9,10]. Isothermal amplification methods enable NA amplification without changes in temperature with simple devices, as well as faster amplification times compared with those of regular PCR.

Loop-mediated isothermal amplification (LAMP) is a well-studied isothermal method, since it provides high sensitivity and specificity with primer sets consisting of four to six specific primers that recognize different regions in the target DNA sequence [11].

The advantages of LAMP include the following: (1) It has higher specificity at constant temperatures over 55 °C with up to three primer sets; (2) it enables rapid reaction times for the exponential accumulation of amplicon; (3) it requires a simple reagent consisting of *Bst.* polymerase, MgSO4, and primers; and (4) it uses simple detection methods, which makesit specific, simple, and POC-friendly.

In addition, the DNA yield rate of LAMP is higher (10 μg/25 μL) than that of traditional PCR (0.2 μg/25 μL) [12].

Moreover, LAMP maintains sensitive and specific amplification, even though the LAMP mixture is reacted with untreated biological fluids that commonly impede PCR-based amplification. Even without an additional process for sample pretreatment and the removal of the inhibitor, LAMP exhibited sensitive detection limitations similar to those of conventional quantitative PCRs [13].

## 2. Principle

**Figure 1:**
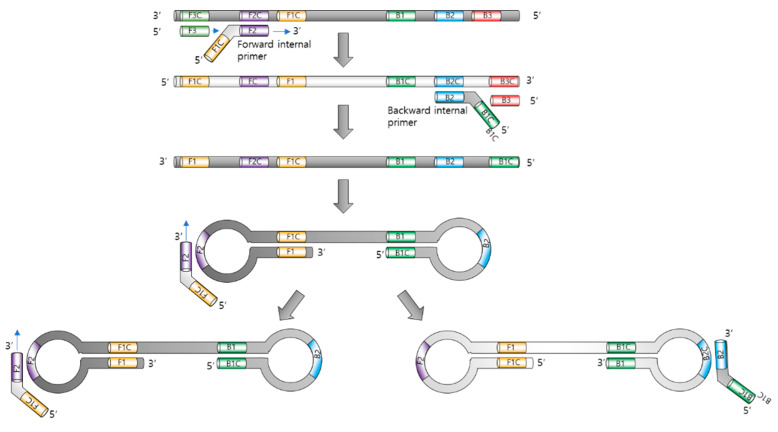
Principle of loop-mediated isothermal amplification. The role of each primer is illustrated.

The principle of LAMP is illustrated in Figure 1. The major components of LAMP are nucleotides, *Bst.* DNA polymerase, primer sets, and a reaction buffer containing magnesium ions.

In LAMP, four to six primers (forward inner primer FIP, forward outer primer F3, backward inner primer BIP, and backward outer primer B3) were used to form loops of DNA replication. The 5′ of FIP is designed to hybridize to the part of the elongated sequence and make a loop. The amplification process uses a single extension temperature, without denaturation or annealing [11].

The inner primers initiate LAMP, and the outer primers release single-stranded DNAs due to strand-displacement DNA synthesis by *Bst.* polymerase. These released DNAs serve as templates for the next inner primers, producing a stem–loop DNA structure.

The *Bst.* polymerase (derived from *Bacillus stearothermophilus*) allows LAMP to unwind DNA strands, resulting in displacement, as with a helicase. In addition, it lacks 5′ to 3′ DNase activity and does not hydrolyze previously synthesized DNA strands, which is an essential property of displacement polymerase.

Initially, the inner primer targeting two different regions in the template target DNA hybridizes with the target DNA, and the polymerase begins to synthesize the complementary strand. Next, the forward outer primer starts to displace the synthesized complementary strand using the forward inner primer, releasing the strand that serves as a template for the backward primers.

The backward inner primer synthesizes the complementary strand, and the synthesized sequence is followed by the displacement of the backward outer primer, similar to that which occurs in the case of forward primer sets. Both the 3′ and 5′ ends were designed to be complementary to the inward sequences for the formation of a loop DNA structure, which is a key factor for exponential amplification.

Self-priming and the elongation of its end induce displacement, the unfolding of the loop, and the refolding of the newly synthesized strand. Repeating the process of self-priming, elongation, and displacement generates long amplicons.

LAMP can be accelerated using additional loop primers [14]. Since LAMP recognizes the target DNA using six different primers, its amplification is expected to be highly selective. The strand displacing the *Bst.* polymerase enables amplification using a normal heating block, avoiding the use of thermal cycling machines.

The primers used for LAMP were designed using online software (Primer-Explorer, http://primerexplorer.jp/e/ accessed on 15 September 2022). However, since the search results do not always guarantee the specificity of the designed primer set, candidate primer sets should be validated using well-designed tests to check whether the primer sets show non-specific reactions.

## 3. Monitoring Methods for LAMP

A major advantage of LAMP is that its amplicon can be confirmed using various simple methods, such as agarose-gel electrophoresis, colorimetric methods with naked eye [15], and real-time fluorimeters [16,17].

Among these methods, the standard methods to confirm presence of LAMP products are the measurement of turbidity [12,18] and colorimetric methods with the naked eye [16,19]. Other methods rely on hydroxy naphthol blue as a metal indicator for magnesium [20], Eriochrome Black T-based colorimetric LAMP [21], coffee-ring effect [22], LAMP–magnetic bead aggregates [23], intercalating fluorescent dyes [24], bioluminescence [25], or electrochemiluminescence [26].

### 3.1. Gel Electrophoresis

**Figure 2:**
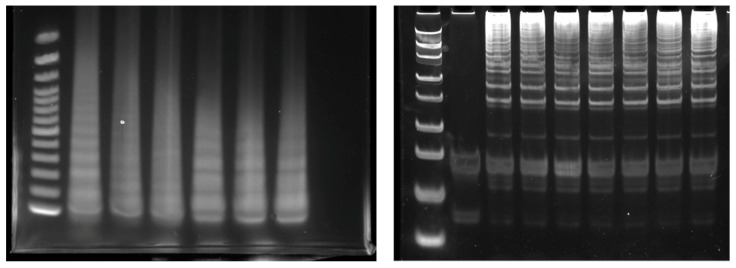
Example of LAMP amplicon monitored with electrophoresis.

Gel electrophoresis is a conventional method for directly monitoring LAMP amplicons [27,28]; LAMP amplicons are visualized and the risk of non-specific detection is reduced. The positive LAMP amplicon subjected to gel electrophoresis appeared in a ladder-like pattern due to the presence of many bands of different sizes [29] (Figure 2). The four basic LAMP primers form two loops at the end of the amplicon, forming a structure similar to a dumbbell, which is then extended. The amplicons appear in a ladder pattern as a consequence of these multiple dumbbell-like structures.

For visualization, the planar group of the polycyclic fluorescent dye was intercalated with the stacked base pairs of double-stranded DNA molecules, resulting in enhanced fluorescence emission. The most common dyes used for checking the LAMP reaction are SYBR Green dye and ethidium bromide [24,28,29,30].

Moreover, microchip-based electrophoresis for the analysis of LAMP products was developed to accelerate monitoring methods for LAMP [31]. However, electrophoresis is only used for qualitative analysis with amplicon of the endpoint. A possible risk of cross-contamination exists because it is performed under open conditions. The requirement for apparatus of electrophoresis and UV detection is a hurdle for POC applications.

### 3.2. Turbidity

During the LAMP-amplification process, the pyrophosphate is released from the deoxynucleotide triphosphate as a by-product, and the pyrophosphate ions react and precipitate with magnesium ions in the reaction buffer [32,33]. Since a large amount of DNA is obtained as the LAMP product, the concentration of pyrophosphate ions is higher than the threshold for precipitation, resulting in a visible precipitate in accordance with the amount of amplicon. The turbidimeter can be used for monitoring the turbidity of the visible precipitate in real time [12,18]. The number of amplicons can also be quantified using a standard curve generated from different concentrations of gene-copy numbers plotted against time to a positive signal [27]. Real-time turbidity-monitoring methods for quantitation do not require a special probe, indicators, or auxiliary reagents and enable high automation.

Wang et al. developed a lab-on-chip device for the rapid, automatic, and sensitive detection of viable *Salmonella typhimurium* using LAMP and smartphone-based real-time turbidity monitoring [34]. The viable bacterial DNA was amplified, and the turbidity of the LAMP product was monitored in real time to quantitatively detect bacteria. The device detected viable *Salmonella* levels as low as 14 colony-forming units (CFUs)/mL in spiked chicken-meat supernatants within 1.5 h.

Wachiralurpan et al. developed rapid and specific LAMP to detect *Listeria monocytogenes* in food and food products using a real-time turbidimeter platform (LAMP turbidity) [35]. After testing the developed device on 200 raw-chicken-meat samples, they demonstrated that the specificity, sensitivity, and accuracy of LAMP turbidity were 100%, 62.75%, and 90.50%, respectively.

Fang et al. developed an eight-channel microfluidic chip to read the product of LAMP by analyzing the turbidity measured using an optic sensor [36]. Using this device, 10 fg of DNA sample was detected.

The main advantage of turbidity monitoring is detection under closed conditions without opening the reaction tubes, making the targets free from cross-contamination during analysis. However, since the turbidity is only stable for a short period [37], the amplicon should be monitored as soon as the LAMP reaction completes. In addition, since the analysis of turbidity is based on a by-product of DNA amplification and by-products are not always directly formed by the specific reaction of a primer, it is not possible to completely exclude non-specific signals, such as salt accumulation, that accompany LAMP reactions, even by a non-primer signal or primer dimers. Thus, if primer dimers non-specifically cause DNA amplification, the increased turbidity can be monitored as a positive signal. The most important part is the validation of the primer set for the LAMP system using turbidity monitoring.

### 3.3. Fluorescence

Generally, the fluorescence-based monitoring of the amplicon of LAMP is reported to be considerably faster (>50%) than that of turbidity-based LAMP [13]. Compared with the real-time turbidity method, the fluorescence-based monitoring method has higher sensitivity and is less affected by the other substances, such as plasmids and proteins, in the mixture [38].

The LAMP monitoring methods that use fluorescent dyes are divided into those that use an intercalating dye and those that modify the primer or probes with fluorescent dyes. Some fluorescent dyes can intercalate into double-stranded structures as the target gene is amplified in the form of double-stranded DNA (dsDNA), resulting in a sensitive optical signal. Hence, these intercalating indicators can be used to monitor LAMP reactions in real time.

**Figure 3:**
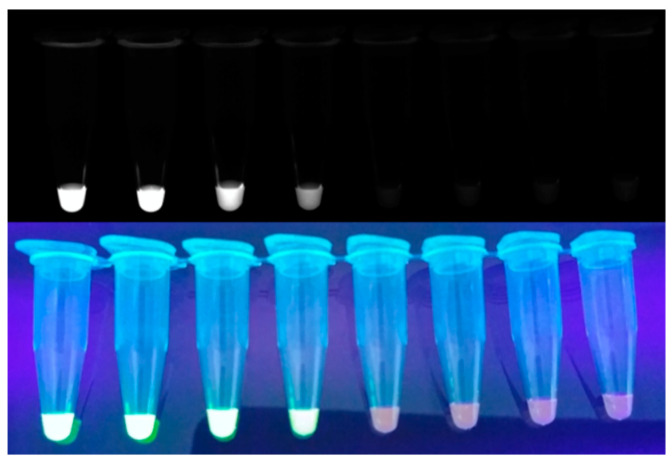
Example of monitoring LAMP amplicon with SYBR Green I.

Since SYBR Green I (Figure 3) is relatively cost effective and quickly intercalates into dsDNA, it is used as a popular intercalating dye for real-time monitoring [39]. Zhang et al. developed a one-step SYBR-Green-I-based RT-LAMP assay to reduce contamination [40]. In this study, they demonstrated that the SYBR-Green-I-based real-time RT-LAMP assay is specific and sensitive for detecting bovine diarrhea virus in biological samples. Kamra et al. developed an SYBR-Green-I-based multi-targeted LAMP (MT-LAMP) assay to diagnose genitourinary tuberculosis [41]. In this study, 55 specimens were evaluated, resulting in 85.5% sensitivity and 94.4% specificity. In addition, the sensitivity of MT-LAMP was significantly higher than that of multiplex PCR. Lucchi et al. developed a portable device capable of performing both the amplification and detection of LAMP on one platform to diagnose malaria [42]. The sensitivity and specificity of the device to detect *P. falciparum* were 96.7% and 91.7%, respectively.

The fluorescent dye calcein has been applied in LAMP [16]. Since calcein is quenched by manganese ions that are deprived of pyrophosphate, the fluorescent signal is emitted when pyrophosphate ions are released during the LAMP reaction.

Wu et al. developed a rapid and efficient method to quantify and differentiate the viable and dead cells of *Salmonella enterica* using ethidium bromide monoazide (EMA) in combination with a real-time LAMP assay [43]. Furthermore, EMA has been used as a DNA-binding dye to differentiate viable and dead cells using PCR [44]. As EMA penetrates only dead cells and DNA that is covalently bound to EMA cannot be amplified by PCR, only DNAs from viable cells are amplified.

Ahmad et al. reported a different fluorescence dye SYTO-82 and a charge-coupled device (CCD)-based imaging system for the rapid detection of water-borne pathogens using LAMP in disposable microchips [45]. The detection system indicated single-copy-level sensitivity for *Campylobacter jejuni* 0414 within 19 min, which is half the time taken by the commercial real-time PCR instrument.

Ohtsuki et al. used YO-PRO-1 iodide to develop a LAMP assay to detect *Brucella* spp. [46]. Stedtfeld et al. used SYTO-81 as a disposable microfluidic chip, which enabled rapid quantitative detection of multiple genetic markers with high sensitivity and specificity based on LAMP [47].

Labeling primers with a fluorescent dye ensures the detection of primer-derived signals only. Wang et al. applied CdSeS/ZnS quantum dots (amine-QDs) as a new reporter probe for LAMP, which enabled single-copy sensitivity [48]. In the presence of the target sequence, the amine-QDs were embedded in magnesium pyrophosphate crystals, leading to their co-precipitation. The limit of detection of the QD-based detection system was 83 zM.

Takayama et al. developed a fluorescence RT-LAMP to detect influenza virus and respiratory syncytial virus (RSV) using a quenching primer (QPrimer) [49]. In this study, QPrimer was labeled with BODIPY FL at the 5′ end. When QPrimer hybridized with its target gene, photo-induced electron transfer occurred between the fluorescent dye and a guanine residue, resulting in quenched fluorescence. Thus, the fluorescence signal reached a maximum at the beginning and was progressively quenched throughout the amplification process. In addition, QPrimer reduced the LAMP detection time, and positive signals were typically detected within 20 min.

Shirato et al. developed fluorescent RT-LAMP assays using quenching probes (QProbes) modified with fluorescent dye at 3′ to monitor primer-derived signals [50]. In the QProbe, since the primer is modified with the fluorescence dye at the 3′ end, the extension of the primer sequence is blocked by the dye. The use of QProbe helps detect only primer-derived signals, and thus avoids detection of non-specific amplification caused by fluorescent primers. The assay was highly specific to Middle-East-respiratory-syndrome coronavirus, showing no cross-reactivity with other respiratory viruses.

Hardinge et al. used the 5′ fluorescent labeling of LAMP primers in LAMP amplification for simple and specific quantification of DNA [51]. By selecting different fluorophores, this method can be extended to multiplexing detection.

Instead of using only the fluorophore, polyethylenimine (PEI), which strongly interacts with DNA, can be used to enhance the fluorescence signals of LAMP. Mori et al. employed PEI to enhance the detection of fluorescent dye-labeled LAMP products [52]. Polyethylenimine neutralized the charged dye-labeled LAMP products, resulting in precipitate formation that could be visually identified.

Fluorescence-based LAMP monitoring methods require a fluorometer; however, portable devices have recently been developed, which enable their use in field surveillance as accurate diagnostic tools.

### 3.4. Naked-Eye Monitoring

Owing to the applicability of many sample types and diverse applications, LAMP protocols are compatible with a wide range of possible indicators that may offer different detection modes and vary in effective concentrations. In addition to gel electrophoresis and fluorescence-based monitoring methods, the process of LAMP can be monitored with the naked eye using various dyes that intercalate with DNA, interact with specific ions, or change the pH. These methods do not incur additional instrumental costs and ease the process of applying LAMP to point-of-care testing (POCT).

#### 3.4.1. Naked-Eye Monitoring with Intercalating Dyes

DNA-binding dyes selectively bind to dsDNA, and the formation of the dye–dsDNA complex causes a visible color change in the dye. The dyes intercalate into the base pairs of dsDNA by stacking the aromatic ring, which leads to changes in the absorption spectra [53]. The change in color is an indicator to qualitatively monitor LAMP. These indicators include leuco crystal violet (LCV) [54], malachite green (MG) dye [15,55], berberine dye [56], and Quant-iT PicoGreen [57].

Firstly, LCV is a colorless dye that is highly selective for dsDNA and does not interfere with ssDNA or deoxynucleotide triphosphates; LCV is obtained from sodium-sulfite treatment with crystal violet (CV). When LCV makes contact with dsDNA, it is reconverted into CV. Therefore, when a target gene is amplified with LAMP reaction, the color of reagents changes from colorless to violet [54].

Secondly, MG is a DNA-intercalating non-fluorescent cationic dye that does not inhibit the amplification efficiency. Pre-addition of MG to the LAMP-reaction solution enabled naked-eye visualization with robust, superior sensitivity and produces reaction products that can be visually distinguished more easily than those produced by existing LAMP fluorescence and turbidity assays. Nzelu et al. applied MG to detect 0.01 parasite of *Leishmania* per reaction, which was more sensitive than that of classical PCR [15]. Lucchi et al. developed an MG-LAMP assay to detect *Plasmodium* spp. with detection limits of 1–8 parasites/μL [58].

Thirdly, berberine was used as an intercalating dye to monitor LAMP with comparable sensitivity to SYBR Green I and EvaGreen [56].

Further, SYBR Green I and EvaGreen can be used as colorimetric and fluorescent indicators because the color of the fluorescent dye SYBR Green I changes from orange to green in the presence of sufficient amounts of dsDNA. However, since high concentrations of dye inhibit LAMP, dyes must be added to the reaction mixture after the amplification process. This means that the reaction tube should be carefully opened after amplification to prevent carry-over contamination. To overcome this drawback, Hong et al. used a tin foil to avoid opening the tubes. In this study, SYBR Green I dye, which was loaded on a tin foil in the tube, was dropped into the LAMP reaction mixture after the LAMP reaction by centrifugation. The limit of detection of tin-foil-assisted LAMP was 1 copy/μL [59].

#### 3.4.2. Naked-Eye-Monitoring Metal Indicator

The LAMP reaction produces a large amount of pyrophosphate ions, which can react easily with Mg^2+^ ions to form precipitating magnesium pyrophosphate. Since the Mg^2+^ ion concentration decreases with the LAMP reaction, Mg^2+^ ion concentration can be used as a LAMP indicator. Based on the reaction with Mg^2+^, various colorimetric indicators, including GeneFinder^TM^ [37], hydroxyl naphthol blue (HNB) [20], Eriochrome Black T (EBT) [21], and calcein [16], have been developed.

**Figure 4:**
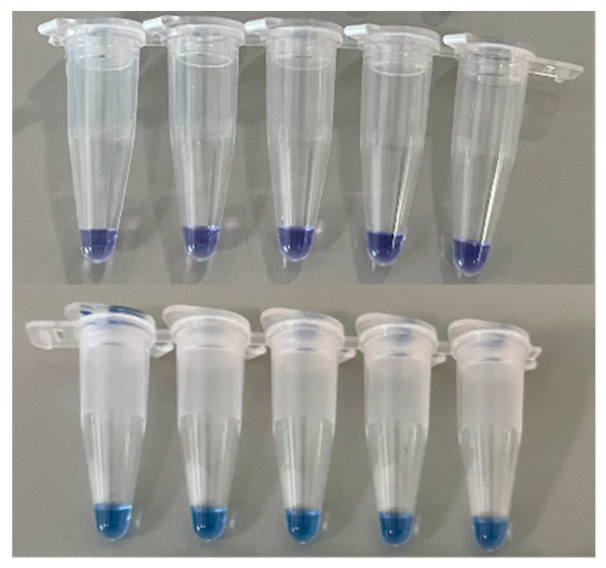
Example of monitoring LAMP with HNB. Upper one is color of tubes before LAMP and the below is after positive LAMP reaction with HNP.

Further, HNB develops a purple color in the presence of Mg^2+^ (Figure 4). As the concentration of Mg^2+^ in the solution decreases during the LAMP process, the color of the HNB solution changes from purple to blue [20]. Furthermore, HNB does not affect the LAMP reaction [57]; hence, it is widely used as a reliable indicator [60,61,62,63,64].

Using HNB, 10^4^ copies of minute viruses of mice were detected; the value was 100-fold lower than that of the conventional PCR [64]. In another case, using HNB-based LAMP, 0.04 pg of *Chlamydia trachomatis* was detected with 95% specificity and 90–100% sensitivity [61].

Hongwarittorrn et al. developed a paper analytical device coated with PEI, which is a strong cationic polymer [65]. Further, HNB reacted with the immobilized PEI to form a visual LAMP sensor. The device used to visually detect LAMP reaction quantified the initial concentration of genomic DNA as low as 4.14 × 10^3^ copies/µL.

The EBT is another metal indicator that causes color change according to Mg^2+^ concentration. As the LAMP progresses, the initial purple color of the LAMP mixture changes to sky-blue. Oh et al. developed a microfluidic system for EBT-LAMP on a chip, which detected Escherichia coli O157:H7 at as low as 380 copies, indicated by color change [21]. For EBT-based colorimetric detection on a chip, EBT was mixed with the LAMP master mixture before the heating process; therefore, the mixture was free from cross-contamination after the LAMP reaction.

The pyrophosphate resulting from the reaction of deoxynucleotide triphosphates with the DNA strand forms a stable complex with bivalent metal ions, such as magnesium, calcium, or manganese.

**Figure 5:**
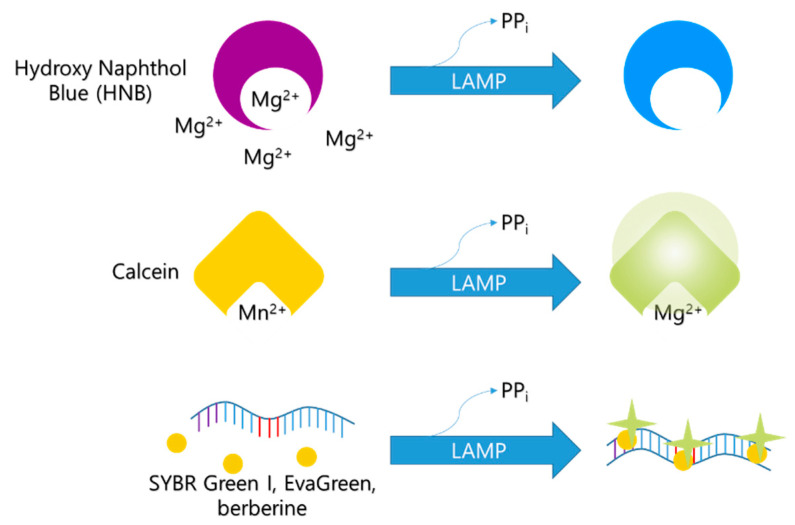
Overview of monitoring method used for LAMP with colorimetric detection.

Before the LAMP reaction, calcein molecules combine with manganese ions, and calcein fluorescence is quenched (Figure 5). The color of the mixture at this stage appears orange. As the LAMP reaction proceeds, manganese ions are released from calcein molecules and combine with the newly generated pyrophosphate ions. The green fluorescence is recovered, and the calcein molecules bind to the residual magnesium ions, enhancing the green fluorescent signal [16]. This change in color can be recognized with the naked eye.

Recently, Lin et al. developed a rapid and visual calcein-LAMP assay targeting the *tetM* gene in *Clostridioides difficile* strains. The detection limit of LAMP was 36.1 pg/µL, which was 100-fold more sensitive than that of PCR [66].

The most important advantage of the metal-binding indicator is that the dye is mixed prior to amplification, and the need to open the sample to add the dye is eliminated. Hence, the risk of contamination is minimized, meaning that the sensitivity of the assay and visual detection in a closed-tube format is well suited to POCT.

#### 3.4.3. Naked-Eye Monitoring with pH Indicator

**Figure 6:**
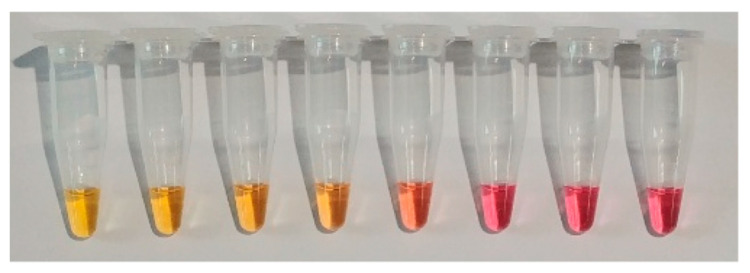
Example of monitoring LAMP amplicon with phenol red.

During the LAMP process, when a DNA polymerase incorporates a deoxynucleoside triphosphate into the DNA, pyrophosphate and hydrogen ions are released. The released hydrogen induces changes in pH, and DNA amplification with LAMP or PCR is monitored using a pH sensor that measures the released protons [67]. Phenol red (PR), cresol red, and neutral red are pH-sensitive dyes that change color as a result of proton generation in a positive reaction [68]. Figure 6 shows one example of color change after LAMP with phenol red. When the positive sample is amplified, the color of the reagents changes to yellow from pink.

Recent reports have demonstrated the accuracy and sensitivity of LAMP using pH-sensitive dyes. A LAMP-based assay that used PR was developed to detect severe acute respiratory syndrome coronavirus 2 (SARS-CoV-2) in 30 min, with a low detection limit of 1000 RNA copies [69]. In another report, a LAMP colorimetric assay that used PR was successfully developed to detect genetic disorders with 100% sensitivity and 98.2% specificity in more than 62 patients; these results were comparable to those of the conventional PCR analysis [70].

As described, the mechanisms of action of indicators vary in the LAMP process. The summary of indicators for monitoring LAMP is listed in Table 1. No gold standard or optimized method exists to monitor LAMP.

Some studies have evaluated dyes in terms of sensitivity, stability, safety, and degree of color contrast based on subjective analysis.

Fischbach et al. compared turbidity, HNB, calcein, SYBR Green I, EvaGreen, and berberine using LAMP primers to detect potato spindle tuber viroid (PSTVd) [56]. According to previous studies, LAMP monitoring methods using HNB, calcein, or berberine are sensitive and can be used in the field.

Chahar et al. compared the efficiencies of HNB, MG, SYBR Green I, and ethidium bromide to detect Pfcrt K76T mutants in *P. falciparum* [71]. As a result of the comparison, the MG- and HNB-based methods were found to be more efficient in terms of time, safety, sensitivity, cost, and simplicity. These two indicators produce a long stable color change and brightness in a closed-tube-based approach to prevent cross-contamination risk.

Scott et al. compared the effects of HNB, PR, LCV, MG, and calcein and demonstrated that LCV and MG provided the best accuracy, followed by HNB and PR [72].

According to the comparative studies, the metal-binding indicator is the most sensitive; however, the decision still relies on subjective interpretation. Hence, digitized quantitative-detection methods have been developed [73].

## 4. Sensing Platform with LAMP

### 4.1. Electrochemical Sensing

Electrochemical methods are used to monitor isothermal amplification products using factors such as selectivity, sensitivity, speed, and cost [74]. The advantage of considering many factors makes electrochemical monitoring the most reliable and efficient method for analyte examination. This section deals with the technology used to monitor LAMP reactions without a large optical device but through electrochemical sensors, chips, and voltammetry devices. The fluorescent staining dye Hoechst 33,258 (H33258) acts as an effective redox probe when it binds to dsDNA. When dsDNA and H33258 are combined, the oxidation peak current is reduced, and this mechanism is used to monitor the LAMP process. Kampeeraa et al. developed a LAMP method with electrochemical detection using an H33258 redox probe and a portable potentiostat for the rapid detection of *V. parahaemolyticus* in raw seafood within 45 min at the detection limit of 0.3 CFU/25 g [75]. Ahmed et al. combined a LAMP amplicon with H33258 on a disposable electrochemical printed chip using linear-sweep voltammetry and detected genetically modified organisms at a low detection limit of 300 copies/reaction [76]. In another study, the electrochemical LAMP was used to detect *Mycobacterium tuberculosis* [77]. The LAMP amplicon was mixed with H33258 immediately after the LAMP procedure, and 50 µL of the mixture were loaded onto the screen-printed graphene electrode to measure changes in cyclic voltammetry for label-free analysis. The in-house potentiostat device indicated a detection limit of 1 pg for the analysis of sputum samples.

Safavieh et al. applied the same LAMP electrochemical principle with H33258 to the microfluidic channel [78]. Using the microfluidic electrochemical sensor, *E. coli* was detected in urine or Luria–Bertani media in 35 min, with a detection limit of 48 CFU/mL.

Methylene blue (MB) is an electroactive molecule that intercalates with the dsDNA. When MB molecules are reduced or oxidized, they transfer electrons to the electrode, resulting in an electrical signal.

Olabarria et al. used MB as an electrochemical redox-active molecule that intercalates with dsDNA to develop a LAMP sensor for the diagnosis of *Legionella* spp. in different water systems [79]. During the LAMP reaction, the MB molecule intercalated with the dsDNA LAMP amplicons, and the electron transfer reaction was reduced, resulting in a decreased electrochemical signal. Using the electrochemical sensor, 10 fg of nucleic acid, which is equivalent to two copies of bacteria, can be detected within 20 min.

Luo et al. developed eight-channel microfluidics for the electrochemical LAMP with MB [80]. In each chamber of the eight channels, the LAMP mixture was loaded, and the LAMP procedure was monitored. With this multi-channel chip, three types of DNA were detected with limits of 28, 17, and 16 copies/μL.

Ramírez-Chavarría et al. applied a LAMP electrochemical sensor to detect SARS-CoV-2 in a real wastewater sample [81]. The LAMP reaction was monitored on a screen-printed electrode using MB. Using this sensor, 2.5 fg/μL of RNA can be measured. However, an additional process involving the pre-concentration and extraction of RNA before the LAMP process is necessary. Developing an all-in-one microfluidic chip that can simultaneously concentrate, extract, and amplify target RNA or DNA is necessary.

**Figure 7:**
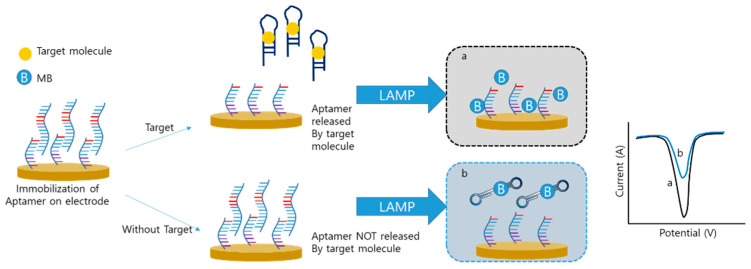
Electrochemical detection of small molecule with LAMP (modified from ref. [82]).

Xie et al. developed an aptamer-integrated electrochemical LAMP sensor to detect small molecules instead of nucleic acids [82]. In this system, the sequence of the aptamer was the same as that of the F3 primer (Figure 7). In the initial step, an aptamer that binds to the main target, orchratoxin A (OTA), was immobilized on the electrode by hybridization with its complementary sequence. In the absence of target OTA, the aptamer was still immobilized on the electrode. The aptamer that remained on the electrode was used for the LAMP procedure, and the MB was intercalated with the LAMP amplicon, which reduced the signal. However, when target and hybridized aptamers are released, the LAMP process does not occur because of the absence of the F3 primer. A high amount of MB results in a high electrical signal. In this study, the LAMP was applied to detect small molecules and the electrochemical sensor was of the “signal-on” type, whereas most other electrochemical LAMP sensors were of the “signal-off” type.

Ruthenium hexaamine (RuHex) binds electrostatically to the anionic dsDNA backbone. Hashimoto et al. developed a microfluidic electrochemical chip to detect miRNAs, which were enzymatically lengthened for amplification with LAMP [83]. The primer was first immobilized on the channel, and then the LAMP mixture, including target miRNA, polymerase, and RuHex as a redox compound, was added to the microfluidic chip. The LAMP process was monitored using the RuHex reaction via linear-sweep voltammetry, and the detection range was 10^3^–10^6^ copies/50 μL.

In electrochemical sensing methods using LAMP, a by-product can be used to monitor LAMP. However, additional time or enzymes are required to convert or hydrolyze these by-products. Xie et al. used the LAMP by-product pyrophosphate to develop an electrochemical detection system [84]. In this system, the main principle is to convert the pyrophosphate to ATP using adenosine 5′-phosphosulfate and ATP sulfurylase. Subsequently, the converted ATP was captured by split aptamer 1 immobilized on the electrode, and the electrochemical signal was enhanced with split aptamer 2 modified with Au@Fe3O4 and MB. This strategy was used for *Nosema bombycis* genomic DNA PTP1 with a low detection limit of 0.47 fg/μL. In another study, the same group used a by-product based on different strategies [85]. By adding pyrophosphatase (PPase) to the LAMP mixture, pyrophosphate (PPi) was hydrolyzed to Pi, which was proportional to the amount of target DNA. Next, pi reacted with molybdate to form molybdophosphate, which is a redox mediator. *Nosema bombycis* genomic DNA PTP1 was detected at a concentration of 17 fg/µL.

Gold nanoparticles (AuNPs) can be used instead of redox molecules, such as MB, RuHex, or H33258. Thayanukul et al. developed an AuNP-based electrochemical sensor to detect *Wolbachia* in mosquitoes [86]. First, captured probes that were immobilized on magnetic beads were pre-hybridized to the reporter probe, which was modified with AuNPs. Next, the pre-hybridized conjugates were incubated with the LAMP amplicon to induce AuNP displacements, which changed the differential pulse anodic stripping voltammetry. The detection limit of this electrochemical sensor is 2.2 fM; however, an additional time of 20 min for pre-hybridization and 30 min for the displacement reaction are required for this electrochemical sensor.

Another electric sensor that applies LAMP is the AC susceptometer. Tian et al. combined LAMP and AC susceptometry to develop a rapid and homogeneous detection system for Zika-virus oligonucleotides [87]. Streptavidin magnetic nanoparticles were pre-mixed with the LAMP reagents, including the analyte and biotinylated primers. The nanoparticles bound to the LAMP amplicons, and their hydrodynamic volumes significantly increased after a successful LAMP reaction. The LAMP detection system could recognize 1 aM synthetic Zika virus oligonucleotide in 20% serum with a total assay time of 27 min.

Compared with optical methods, electrochemical methods are more stable and accurate [80]. In addition, according to a study that compared 10 redox reporters [88], the strongest dsDNA intercalators were MB and its derivative, and these intercalators remained unaffected by the inhibition of the LAMP reaction.

### 4.2. Lateral-Flow-Assay-Based Sensing

**Figure 8:**
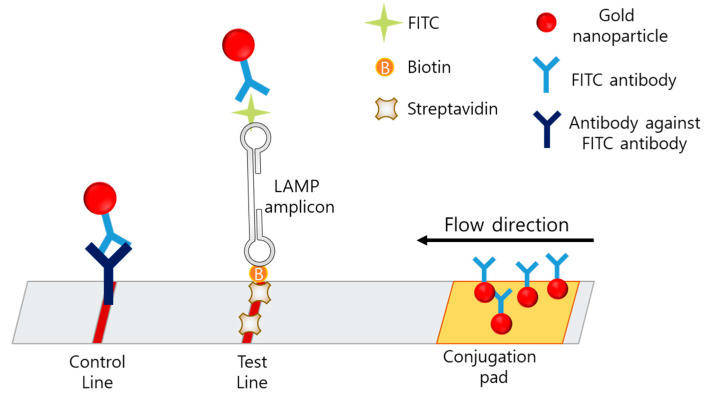
The example of LFA based LAMP sensor.

The lateral-flow assay (LFA) is a simple, cheap, user-friendly, and portable method based on an immunochromatographic technique and is the most favored method for POCT. In the case of LAMP–LFA, after the LAMP process, the LAMP mixture is loaded on the LFA strip, which consists of a conjugation pad, test line, and control line (Figure 8).

In LAMP-LFA, primers are modified with fluorescein isothiocyanate (FITC) and biotin [89,90]. The strip consists of a conjugation pad with AuNP-labeled anti-FITC, a test line with anti-biotin or avidin, and a control line with antibodies against anti-FITC. After the LAMP process, the amplicon is modified with FITC and biotin, based on the primer modification. When the amplicon passes through the conjugation pad, AuNPs labeled with anti-FITC are bound to the amplicon, and the amplicon is captured by avidin on the test line. If no LAMP amplicon is present, the AuNP-labeled anti-FITC is passed through the test line and captured on the control line by the anti-FITC antibody.

The use of an example protocol, 30 min for the LAMP process and 10 min for the visualization of the amplicons, makes the analysis simple. When nucleic acids were extracted for 10 min, LAMP-LFA detection resulted in a total assay time of approximately 50 min. Huang et al. used this protocol and detected the *Karlodinium veneficum* ITS gene (toxic bloom) with a detection limit of 7.4 pg/mL; the value represents sensitivity that is 10 times more than that of conventional PCR [89]. The same group detected *Skeletonema costatum* (toxic bloom) with a detection limit of 0.94 pg/µL; the value represents sensitivity that is 100 times more than that of conventional PCR [90]. Mei et al. used a similar protocol to detect the Salmonella *hilA* gene with a detection limit of 13.5 fg/μL; the value represents sensitivity that is 1000 times more than that of conventional PCR [91].

**Figure 9:**
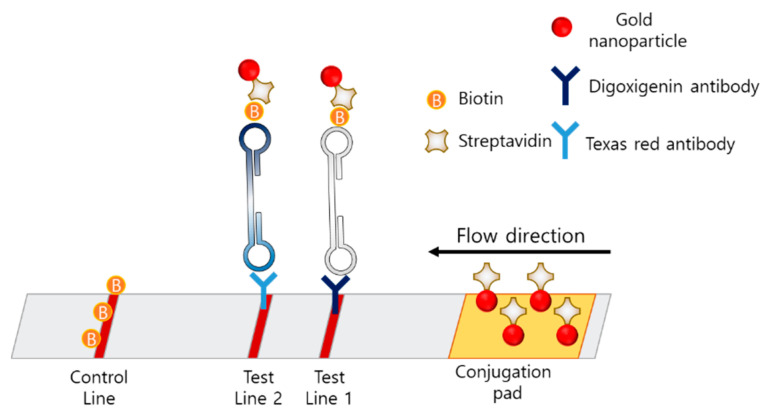
Example of multiplexing LFA for LAMP amplicons.

Multiplexing techniques using LAMP–LFA strips have also been reported. The concept of the multiplexing LAMP is illustrated in Figure 9. Multiplexing in LFA was enabled by dispensing different types of antibodies on the two test lines. Park et al. developed a disk-type microfluidic chip for LAMP–LFAs [92]. The strips were integrated in the disk for an all-in-one-type POCT. The strip consisted of a conjugation pad with streptavidin-coated AuNPs, test line 1 with digoxigenin antibody, test line 2 with Texas Red, and a control line with biotin. The LAMP amplicon was designed to contain biotin, digoxigenin, or Texas Red, according to the target gene. Using this disk, *Salmonella typhimurium* and *Vibrio parahaemolyticus* were detected with a detection limit of 50 CFU in 80 min from extraction to signal reception. Zhu et al. developed a LAMP–LFA for SARS-CoV-2 consisting of a conjugation pad with streptavidin-modified AuNPs, test line 1 with anti-FITC antibody, test line 2 with anti-digoxin antibody, and a control line with biotin [93]. Since the primers were modified with FITC, digoxin, or biotin, the amplicons were also labeled with FITC, digoxin, or biotin. The limit of detection was 12 copies per reaction, and both sensitivity and specificity were 100%.

Multiplexing LAMP–LFA can be used as an internal control to prevent false-negative results by proving that the LAMP process is successfully performed with an additional primer set for plasmid DNA [94]. With the internal control included in the same tube, the reliability of each reaction is significantly increased, and additional separate tubes for the positive control are not required. Najian et al. used multiplexing LAMP–LFA to simultaneously amplify the internal-control plasmid. The LAMP reaction was processed with two types of primers, the internal control and the main target, and the amplicon was loaded on the strip with two test lines and a control line. With the multiplexing LAMP–LFA, 0.3 copies/mL of the genomic DNA of the *Leptospira LipL32* gene were detected.

Further, the LAMP–LFA is a specific and sensitive method that does not require any special instruments for analysis. Hence, it is considered the best candidate for POCT. McConnel et al. developed a LAMP–LFA prototype to diagnose hepatitis C virus that fulfils the World Health Organization guidelines [95]. In this prototype, the LF primer was modified with biotin, and the LB primer was modified with FITC. The strip consisted of a conjugation pad with AuNP-labeled streptavidin, a test line with an anti-FITC antibody, and a control line with biotin. Using this prototype, which can simultaneously analyze four strips, 398 copies can be detected within 40 min.

With tube-type colorimetric monitoring methods, such as HNB and MG, it is not easy to design the multiplexing in a single tube. The important advantage of LAMP–LFA is that it makes it possible to design multiplexing LAMP-LFA. However, time-consuming processes for the pre-loading of antibodies or AuNPs and the instability of immobilized antibodies for long-term storage should be considered before applying LAMP–LFA to POCT.

### 4.3. Optical Sensing with LAMP

Raman scattering was first experimentally observed in 1928 [96]. Even though the scattered Raman spectrum can be used to identify specific molecules inside a mixture, the phenomena cannot be practically applied because the signals are not sufficiently strong. Surface-enhanced Raman scattering (SERS) has been used in various fields because of the strong enhancement of the Raman signal on silver electrodes [97].

**Figure 10:**
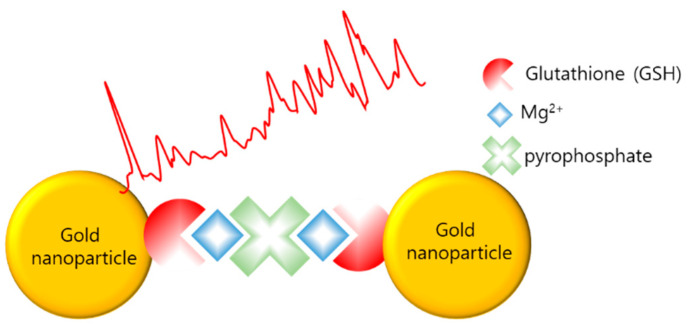
SERS with gold aggregation induced by pyrophosphate, LAMP byproduct (modified from [98]).

Teixeira et al. reported the detection of LAMP amplicons using SERS (LAMP–SERS) for Listeria monocytogenes [98]. As illustrated in Figure 10, LAMP–SERS uses the LAMP by-products, magnesium and pyrophosphate ions, to aggregate AuNPs, which enhance Raman scattering. Additionally, AuNPs were functionalized with 1-naphthalenethiol as a Raman reporter and with glutathione to chelate magnesium and thiolated polyethylene glycol for stabilization. The functionalized AuNPs aggregated due to the complexation of pyrophosphate and glutathione with free magnesium ions when the target gene was amplified during the LAMP process. Using LAMP–SERS, 102 pg/µL of Listeria monocytogenes were detected; the value represents sensitivity that is 10 times more than that of turbidity-based detection.

**Figure 11:**
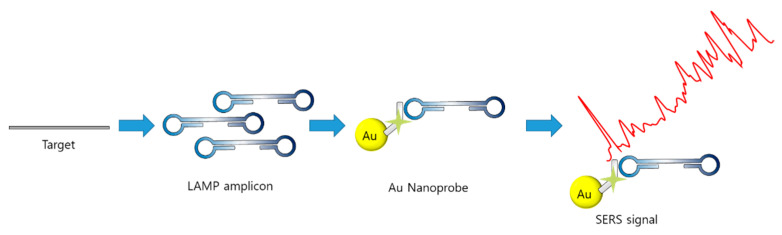
Example of SERS-based detection of LAMP amplicon (modified from [99]).

Dras et al. implemented probe DNA and nuclease in the SERS–Raman technique [99]. After the LAMP reaction, the thiolated probe–AuNP conjugation was added to the LAMP reagents to capture the target LAMP amplicon on the AuNPs. The probe was internally labeled with a Raman reporter cy5 at the sixth base pair for a distance of less than 2 nm from the AuNP, enhancing the Raman scattering (Figure 11). After capturing the amplicon, the mixture was treated with DNA nuclease, which specifically digests ssDNA. In the absence of the target, the ssDNA probe was digested, and cy5 did not enhance the Raman signal. Using SERS–Raman, *Salmonella enterica* (foodborne pathogen) was detected with a limit of 66 CFU/mL, which is 100 times higher than that of the conventional PCR method.

Surface plasmon resonance (SPR) sensors are optical sensors that detect interactions on a surface by measuring changes in the refractive index in real time [100]. The binding event between the analytes and recognition molecules on the sensor chip induces a change in the mass on the surface, which is transduced to a change in the angle. SPR sensors have been applied in various fields to measure binding events. Furthermore, LAMP amplicons can be analyzed using SPR [101]. The LAMP amplicon is hybridized to the probe on the sensor chip, and AuNPs labeled with another probe are added to enhance the SPR signal. The detection limit of the SPR–LAMP sensor for methicillin-resistant *Staphylococcus aureus* in clinical samples was 10 copies/mL. Despite its low detection limit, implementing the SPR–LAMP technique for POCT is challenging because of its price and portability.

Optomagnetic sensors measure the dynamic behavior of magnetic nano-beads (MNB). The interaction of the target analytes and recognition molecules leads to a sensitivity change in the dynamics of the MNB. Since Donolate et al. first reported an optomagnetic sensor with a 405-nanometer laser [102], various fields have applied optomagnetic sensors to analyze the interactions of biomolecules. Tian et al. used biotin-labeled primers for the LAMP amplification of the Newcastle disease RNA virus [103]. The biotin-labeled amplicon bound to the MNB increases the hydrodynamic volume of the MNB and changes its optomagnetic spectra. The limit of detection of the sensor for the RNA virus of Newcastle disease is 10 aM; this value represents sensitivity that is comparable with that of real time-PCR. Minero et al. developed an optomagnetic sensor to detect the LAMP amplicons of dengue virus [104]. The amplicons are captured using sequence-specific hybridization, which enables true positives and false positives to be distinguished by comparing melting temperatures. With the optomagnetic sensor, 100 fM of dengue virus can be detected within 20 min of the LAMP reaction.

Another optical sensor uses special particles that blink according to the viscosity of the suspension. Das et al. integrated LAMP with a particle-imaging technique to sensitively measure the SARS-CoV-2 gene without dyes or indicators [105]. The large number of amplicons changes the viscosity of the LAMP solution, and when Janus particles are added, a blinking signal is produced that is inversely proportional to the viscosity. The limit of detection is 70 ag/μL, the required sample volume is 2 μL, and the analysis time is 10 min. This technique still requires a fluorescent microscope and a CCD camera.

The LAMP amplicon can be monitored using flow cytometry [106]. In this study, to measure the hepatitis C virus (HCV) with LAMP using flow cytometry, the FIP primer was immobilized on the magnetic beads through biotin–streptavidin interactions, and the BIP was modified with Cy5. Before the LAMP assay, FIP-immobilized beads were incubated to capture the target and mixed with other LAMP reagents. The reagent was then divided into small droplets by the formation of water in an oil emulsion. The targets and primers in the droplets reacted during the LAMP process. After the emulsion LAMP reaction, the beads were analyzed using flow cytometry to count the HCV copies in the sample. Using this digitized LAMP reaction and flow-cytometric analysis, 300 copies of HCV were detected without any additional chips to analyze the separated target in a droplet.

An ideal monitoring sensor should be highly sensitive, non-labor-intensive, user-friendly, environmentally friendly, feasible, swift to respond, and of low cost, both in the laboratory and at the point of care. As described above, the LFA-based sensing platform is the best in terms of mobility and portability. However, the strip-preparation process is complicated, and the stability of the antibodies should be considered. The optical sensor requires complicated equipment, such as a turbidimeter, SPR sensor, fluorimeter for flow cytometry, and Raman spectroscopy. Electrochemical sensors are portable with a miniaturized potentiostat that does not compromise sensitivity.

## 5. Devices to Monitor LAMP

As smartphones are emerging as the most promising monitoring devices, providing portability, affordability, and rapidity, they can be used as readout [107,108,109,110], image-processing [111,112], or operating tools [55,113] on diagnostic platforms. Furthermore, LAMP has been applied in diagnostic systems using smartphones owing to its various monitoring mechanisms, including color change, fluorescence, and turbidity, which can be implemented on smartphones.

Recently, Song et al. reported a smartphone application based on machine learning for checking diagnostic results [111]. The diagnosis of SARS-CoV-2 and its variants consists of a colorimetric DNAzyme reaction triggered by a LAMP amplicon with clustered regularly interspaced short palindromic repeats (CRISPR). The CRISPR was used to eliminate false-positive LAMP amplicons and improve the accuracy. With smartphone-based diagnostics, an attomolar sensitivity range was achieved.

Diego et al. developed a handheld device for LAMP that was controlled by a smartphone application [55]. The device was designed for the real-time colorimetric monitoring of the LAMP reaction. Zhou et al. developed paper fluidics on which LAMP amplification was enabled to detect *Escherichia coli* O157:H7 [113]. Smartphones can control the temperature of the module and collect fluorescent images. After collecting images with a smartphone, the RGB value was extracted from the fluorescence images of the paper fluidic chip using the android app, “Color Picker”. Using this smartphone-based diagnostic, 28 fg/μL of the target was detected. Nguyen et al. developed smartphone-based LAMP diagnostics to detect SARS-CoV-2 [114]. On the microfluidic chip, detection processes, including the extraction of nucleic acids, LAMP amplification, and the monitoring of the fluorescent signal, were automatically performed. The fluorescence intensity was measured and transferred to a smartphone in real time. Using an all-in-one microfluidic chip, 20 copies/μL of SARS-CoV-2 were detected. Papadakis et al. developed a portable device for colorimetric LAMP that can be operated using a smartphone [73]. Using this device, a real-time color image of the sample tube can be captured during the LAMP process using the vertical design of the imaging camera. Furthermore, five copies of the target gene can be detected. When this device was tested with extracted coronavirus disease 2019 RNA, the sensitivity was 97%.

Smartphones are convenient and simple image-reading tools. Xie et al. developed a multiplexing LAMP device with calcein fluorescent signals and used a smartphone for image acquisition [109]. For fluorescence excitation, the samples were illuminated with an additional 470-nanometer LED. Kaarj et al. developed a wax-printed paper microfluidic chip to detect Zika virus using LAMP and used the pH indicator, phenol red, for monitoring [110]. The color change of the LAMP amplicon was monitored using a smartphone camera, and the images were evaluated using Image J software. By analyzing the image taken by the software, one copy/μL of Zika virus was detected.

Recently, the self-testing of LAMP reactions has become possible with the availability of commercialized Lucira™ and Detect™ at home. The results can be transferred to a smartphone application. In the case of Lucira™, the color change of the reaction mixture induced by a change in pH was detected using optical and electronic elements in the test unit. The clinical sensitivity was 92% and the clinical specificity was 97%. In the case of Detect™, after LAMP amplification in Detect Hub™, the reaction tube was inserted into the reader, and the results were monitored on the lateral-flow strip. The clinical sensitivity was 90.9% and the specificity was 100%.

## 6. LAMP Assays for SARS-CoV-2

Since the outbreak of SARS-CoV-2, many diagnostic methods have been developed, including PCR and LFA. Although PCR is the most accurate and sensitive method, it requires specialized equipment and laboratories. Moreover, detecting the surface proteins of viruses using LFA is rapid and inexpensive. However, LFA yields false signals and is much less sensitive and specific compared with molecular diagnosis. Hence, LAMP-based diagnosis is an effective alternative with which to detect SARS-CoV-2. As mentioned above, LAMP requires simple equipment for heat treatment, and the reaction time is usually less than an hour.

For the LAMP assay, reverse transcriptase was used to prepare cDNA using RNA [115]. Subsequently, the cDNA of a specific gene was amplified with primers, and the amplification was monitored using various methods. Target genes can be conserved regions, such as the *ORF1ab* gene, which replicates the genome, the S gene, which encodes the surface protein that interacts with the ACE2 protein, and the N gene, which encodes nucleocapsid proteins.

Yu et al. developed iLACO [116], which implemented the pH colorimetric indicator SYBR Green I and GeneFinder to monitor the LAMP amplicon of the *ORF1ab* gene. The detection limit was 60 copies/µL.

In another study, the LAMP was integrated into the sequencing platform for SARS-CoV-2 RNA in LAMP-Seq [117]. Without any extraction processes, the lysates can be directly used in the sequencing platform. After sterilizing the lysates at 95 °C, 8.3 µL of the lysed sample were loaded onto a 384-well plate. The plates were heated at 65 °C with the LAMP reagent, including FIB labeled with a barcode, and sequenced with MiSeq equipment. The limit of detection was approximately 2.2 molecules per µL.

Song et al. integrated two types of isothermal amplification [118]. LAMP was used after recombinase isothermal amplification (RPA) to enhance the diagnostic sensitivity. The integrated diagnostic method was 10 times less sensitive (five copies pre-reaction) than normal LAMP.

**Figure 12:**
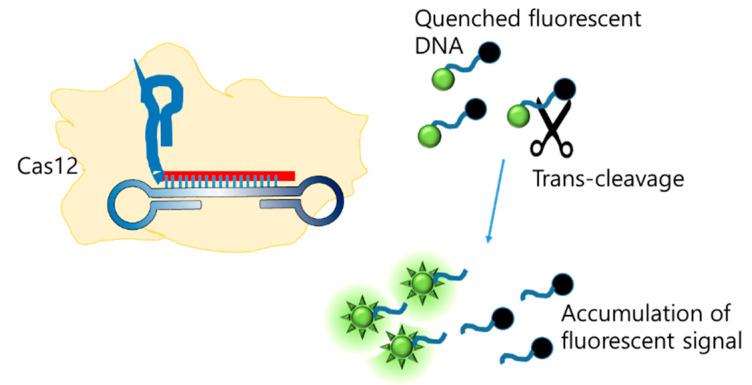
Enhancing LAMP signal with CRISPR/Cas trans-cleavage (modified from [120]).

Another integration method involves combining LAMP with CRISPR/Cas. Since the discovery of Cas12 [119], which cleaves non-specific ssDNA when activated, CRISPR/Cas has emerged as a tool for the generation of new molecular diagnoses. Broughton et al. integrated LAMP with CRISPR/Cas to detect SARS-CoV-2, and the final detection was visualized on an LFA [120]. Further, CRISPR consists of gRNA, which recognizes the LAMP amplicon as a target, and the Cas protein, which non-specifically cleaves the ssDNA probe (Figure 12). The cleaved probe that was labeled with FAM was captured by the FAM antibody loaded on the test line. The limit of detection was 10 copies/µL.

Further, a multiplexing LAMP was developed. Jang et al. reported a primer set to detect the RNA-dependent RNA polymerase (*RdRP*), *E* gene, and *N* gene in SARS-CoV-2 [121]. The authors compared the primer sets and concluded that the primer combination with *RdRP*, *N*, and internal control provided reliable sensitivity compared with that of commercial RT-PCR kits, such as Allplex™ 2019-nCoV Assay (Seegene) and PowerChek™ 2019-nCoV Real-Time PCR Kit (Kogenebiotech).

Furthermore, LAMP can be used to amplify salivary RNA. Taki et al. developed RT–LAMP to detect SARS-CoV-2 in saliva, which indicated sensitivity and specificity of 97% and 100%, respectively, with RNA extraction [122].

## 7. Conclusions

In this review paper, the principles of LAMP and the mechanisms and examples of the methods used to monitor its results were discussed. Subsequently, the principles and examples of biosensors that use LAMP, such as optical, electrochemical, and LFA sensors, were described. Furthermore, examples of the development of smartphone-based POCT technology using LAMP and cases of LAMP-based self-diagnosis were examined. Finally, a representative example of LAMP technology for the detection of SARS-CoV-2, which has recently become the greatest medical challenge worldwide, was discussed.

The usefulness and effectiveness of LAMP have been proven in many studies. Furthermore, PCR-based diagnosis is the standard method to diagnose infectious diseases, such as SARS-CoV-2. However, the development of simpler, cheaper, and faster molecular diagnostic technologies using LAMP will create new molecular-diagnosis standards. Further, LAMP-based molecular diagnosis can replace PCR technologies, which are limited to central and private laboratories in developed countries.

## Figures and Tables

**Table 1:** Summary of indicators for monitoring LAMP.

	Indicator	Cross Contamination	Adding Moment	Detection Mechanism
Magnesium pyrophosphate	Turbidity	No	Not adding	Insoluble precipitant
SYBR Green I	Color to green	Possible	After LAMP	Intercalation
Hydroxy Naphthol Blue (HNB)	Color to blue	No	Before LAMP	Metal binding
Calcein	fluorescent	No	Before LAMP	Metal binding
Malachite Green	Color to blue	No	Before LAMP	Metal binding
Phenol Red	Color to yellow	No	Before LAMP	pH indicator
